# Surgeons’ practices and preferences for performing right hemicolectomy for colorectal cancer: results from the SuPrHem survey

**DOI:** 10.1007/s13304-026-02523-y

**Published:** 2026-02-11

**Authors:** Pierre-Alain Tokoto, Elin Meyer, Eleftherios Gialamas, Guillaume Meurette, Emilie Liot, Justin Davies, Frédéric Ris, The SuPrHem Collaborative Group, Ahmed Abdelsamad, Liza Abraham, Belkacem Acidi, Dmitry Adamovich, Alberto Aiolfi, Abdulahad Al-Ameri, Omar Al Natour, Mohammed N. AlAli, Fatema Albastawis, Syed Muhammad Ali, Asif Ali Amir Ali Khowaja, Turki Alshammari, Chiara Amatucci, Easan Anand, Stefano Andrianello, Elissavet Anestiadou, Vaihere Delaune Anez, Alexandros Chamzin Angélopoulos, Sobhana Iftekhar Tani Anwar, Josué Espinosa Apezteguia, Alberto Arezzo, Alazar Berhe Aregawi, Claudia Armellin, Gabriela Aracelly Arroyo Murillo, Oktar Asoglu, Azharuddhin Attar, Goran Augustin, Bausys Augustinas, Ninos Ayez, Andrea Balla, Alexandre Balaphas, Caterina Baldi, Christos Barkolias, Suman Baral, Ernesto Barzola, Somprakas Basu, Mr George Bazoua, Vittoria Bellato, Andrea Belli, Filippo Banchini, Mehmet Sah Benk, Stefan Benz, Alazar Berhe Aregawi, Federica Billeci, Francesco Bianco, Clarisa Bîrlog, Stephan Bischofberger, Slavomir Blazej, Luca Domenico Bonomo, Pedro Botelho, Marylise Boutros, Graham Branagan, Giuseppe Brisinda, Marco Brizzolari, Walter Brunner, Nicolas C. Buchs, Rory Callan, Sergio Calandra, Giacomo Calini, Marco Cannistra, Gabriella Teresa Capolupo, Filippo Carannante, Fabio Carbone, Marco Caricato, Gianluca Cassese, Antonio Castaldi, Marco Catarci, Gianmaria Casoni Pattacini, Antonio Caycedo-Marulanda, Francesco Ceci, Giovanni Cestaro, Mohamed Ali Chaouch, Georgios Chatziantoniou, Floryn Cherbanyk, Andrei Chitul, Christos Chouliaras, Prokopis Christodoulou, Niki Christou, Enrico Ciferri, Roberto Cirocchi, Nicola Colucci, Diego Coletta, Renan Colombari, Luigi Eduardo Conte, Guido Coretti, Rogier Crolla, Panagiota Dalla, Spyridon Davakis, Gerald David, Giuseppe Massimiliano De Luca, Pierre-Yves De Queiroz, Belinda De Simone, Norma Depalma, Giacomo Deiro, Vaihere Delaune, Federica Di Marco, Jehangir Diyani, Luca Domenico Bonomo, Jonathan Douissard, Liviu Draghici, Bogdan Dumbrava, Audrisu Dulskss, Arnaud Dupuis, Ahmed Elsheikh, Lorenzo Epis, Chiara Fantozzi, Marryam Riaz Farooqui, Alessia Fassari, Ben Finlay, Tahar Fillali, Guido Fiorentini, Gaetano Florent, Gaetano Florio, Caterina Foppa, Alain Foxius, Pietro Fransvea, Francesco Ferrara, Alice Frontali, Raffaele Galli, Cristián Gallardo, Rafael Garamea, Zoe Garoufalia, Rafael Garatea Grau, Georgios Garopoulos, Francisco Gaticia, İbrahim Ethem Gecim, Georgios Geropoulos, Eleftherios Gialamas, Marco Giacometti, Giuseppe Giuliani, Antonio Giuliani, Mario Giuffrida, Ugo Giustizieri, Carlos Augusto Gomes, Camila Gomes, Carmen Loredana Gorgan, Lorenzo Gori, Ramu Gopi, Florin Grama, S. Granger, Umberto Grandi, Rafael Garatea Grau, Pasquale Gualtieri, Francesco Guerra, Bhaskar Guharoy, Mohamed K. F. Hamed, Nir Horesh, Karoline Horisberger, AlAnbat Huda, Basim Hussein, Noelia Ibáñez, Sobhana Iftekhar Tani, Inan Iksan, IHSAN Inan, Orestis Ioannidis, Pouya Iranmanesh, İlgar Ismayilov, Matas Jakubauskas, Aeris Jane D. Nacion, Jin Jiun Mah, Heman Joshi, Maximilian Jusufi, Panagiotis Kapsampelis, Lysandros Karydakis, Ioannis Katsaros, Andrea Kazemi Nava, Deborah S. Keller, Raja Basit Khan, Asif Ali Amir Ali Khowaja, Rémy Kohler, Elissaios Kontis, Georgios Korovesis, Alexandros Kozadinos, Philippe Koppes, Bojan Kovacevic, Igor A. Kryvoruchko, Marius Kryzauskas, Sanjeev Kumar, Naveena A. N. Kumar, Eleandros Kyros, Vasilios Kymakis, Harish Neelamraju Lakshmi, Lorenzo Latham, Joël L. Lavanchy, Emilie Liot, Alexis Litchinko, Antonio Luberto, Andrea-Pierre Luzzi, Hemendra Mangal, Faiza Malik, Carlo Alberto Manzo, Alessandra Marano, Nirvana Maroni, Angelo Alessandro Marra, Sleiman Marwan-Julien, Giuseppe Massimiliano De Luca, Jakubauskas Matas, Luca Mazza, Gennaro Mazzarella, Alexandra Menni, David Merlini, Jeremy Meyer, Zohaib Mian, Nikolaos V. Michalopoulos, Almir Miftaroski, Erineos Michaelides, Nikolaos Michalopoulos, Valentina Miacci, Pamela Milito, Uroš Milošević, Yevgen Miroshnychenko, Ahmed Mohammed Ali ALHaaj, Mohamed Mohammed, Igor Monsellato, Mauro Montuori, Gabriela Aracelly Arroyo Murillo, Adam Mylonakis, Aeris Jane D. Nacion, Surennaidoo Naiken, Gustavo Nari, Muhammad Nasser, Milica Nestorovic, Guglielmo Niccolò Piozzi, Guglielmo Nicolò Piozzi, Antonella Nicotera, Aristotelis Nikitaras, Aristotelis Nikitaras, Ayez Ninos, Nuri Okkabaz, Alba Oliva, Stefano Olmi, Pim B. Olthof, Lorenzo A. Orci, Monica Ortenzi, Paolo Ossola, Vincenzo Papagni, Paolo Panaccio, Andreas Panagakis, Eleni Papamattheou, Dimitra Papalouka, Francesco Pata, Elena Patanè, Hugh Paterson, Alberto Patriti, Nikolas Pauk, Corrado Pedrazzani, Gianluca Pelino, Andrea Peloso, Rajeev Peravali, Slobodan Peric, Bruno Perotti, Georgios Peros, Lorenzo Petagna, Filippo Petrelli, Thalia Petropoulou, Biagio Picardi, Arcangelo Picciariello, Enrico Pinotti, Guglielmo Niccolò Piozzi, Brunella M. Pirozzi, Amandeep Pooni, Mark Potter, Gabriele Pozzo, Vicent Primo Romaguera, Sami Rafiq, Hossein Rahmati roudsari, Michaela Ramser, Gopi Ramu, Maria Chiara Ranucci, Emeka Ray-Offor, Elisa Reitano, Ieuan Reece, Martin Reichert, Marryam Riaz Farooqui, Frederic Ris, Miguel Rivera, Dario Ribero, Mateo Rios, Marryam Riaz Farooqui, Frederic RisMiguel Rivera, Dario Ribero, Fausto Rosa, Maurizio Romano, Vicent Primo Romaguera, Raffaello Roesel, Mirza Faraz Saeed, Kapil Sahnan, Panagiotis Sakarellos, Matteo Santoliquido, Giulio Santoro, Cosimo R. Scarpa, Carlo Alberto Schena, Boris Schiltz, Marcel André Schneider, Dimitrios Schizas, Ana Seara, Angelo Searo, Bruno Sensi, Niyaz Shadmanov, Shafaque Shaikh, Noam Shussman, Peter Siaperas, Bouyagui Sissokho, Leandro Siragusa, Alistair A. P. Slesser, Maria Sotiropoulou, Claudio Soravia, Moritz Sparn, George Stavrou, Jasper Stijns, Sentilnathan Subramaniam, Baral Suman, Athanasios Syllaios, Athanasios Syllaios, Tamas Sztipits, Lucio Taglietti, Giovanni Tebala, Antoine Thicoipe, Sobhana Iftekhar Tani, Giovanni Tomasicchio, Victor Tomulescu, Desmond P. Toomey, Olivier Tobler, Paola Tramelli, Κonstantinos Tsimogiannis, Florin Turcu, Dorottya Turu, Elizabeth Tweedle, Georgios Tzikos, Mustafa Yener Uzunoglu, Michail Vailas, George van der Schelling, Gabrielle H. van Ramshorst, Carsten T. Viehl, Danilo Vinci, Hamza Waqar Bhatti, Rauf Wani, Charles-Henri Wassmer, Steven Wexner, James Wheeler, Luke Wheldon, Omer Yalkin, Mustafa Yener Uzunoglu, Muhammad Umar Younis, Matteo Ζanello, Jeremy Meyer

**Affiliations:** 1 Division of Digestive Surgery, Department of Surgery, University Hospitals of Geneva, Rue Gabrielle-Perret-Gentil 4, 1211 Geneva 14, Switzerland; 2 Medical School, University of Geneva, Rue Michel Servet 1, 1205 Geneva, Switzerland; 3 Cambridge Colorectal Unit, Addenbrooke’s Hospital, Cambridge University Hospitals NHS Foundation Trust, Cambridge, UK; 4 University of Cambridge, Cambridge, UK

**Keywords:** Robotic, Laparoscopy, Minimally invasive surgery, MIS, Colorectal cancer

## Abstract

Surgical techniques for right hemicolectomy (RHC) show considerable heterogeneity. To identify potential areas for improvement and standardization, the objective of this survey was to evaluate and report current practices and preferences among surgeons for performing RHC for colorectal cancer. An international online survey consisting of 31 questions was distributed to general surgeons worldwide to assess their preferred techniques for performing RHC. A total of 368 surgeons from 48 countries responded. Among them, 47% reported performing at least 90% of RHC laparoscopically, while 40% used a robotic approach. The most common dissection technique was a medial-to-lateral vessel-first approach, used by 82.3% of respondents. During laparoscopic procedures, 51.6% of surgeons performed extracorporeal anastomosis, whereas 82.9% of those using a robotic approach favored intracorporeal anastomosis. Fluorescence angiography was routinely used by 39.9% of respondents. The Pfannenstiel incision was the most frequently selected extraction site (46.5%) especially if intracorporeal anastomosis was performed (81.8%). The preferred technique for performing RHC includes a laparoscopic approach with intracorporeal lymph node dissection and extracorporeal anastomosis. Most RHC procedures therefore remain laparoscopy-assisted rather than fully minimally invasive. Robotic surgery appears to facilitate a fully minimally invasive approach including intracorporeal anastomosis.

**Supplementary Information:**

The online version contains supplementary material available at https://doi.org/10.1007/s13304-026-02523-y.

## Introduction

In the United States, more than 250,000 colectomies are performed annually to treat various colonic diseases, including cancer, inflammatory bowel disease and diverticulitis [2]. Right hemicolectomy (RHC) accounts for approximately 40–45% of colorectal resections performed for colorectal cancer [1]. It is often among the first subspecialized procedures undertaken by surgeons in training.

Recent advancements in surgical techniques and perioperative management—including the adoption of robotic-assisted surgery [3], fluorescence angiography [4], complete mesocolic excision (CME), enhanced recovery after surgery (ERAS) protocols [5], and the use of preoperative oral antibiotics [5, 6] have introduced considerable heterogeneity in practice. This variability affects not only the surgical approach but also key technical aspects such as colonic mobilization, lymphadenectomy technique and extent (D2 vs. D3), ileocolic anastomosis creation, and selection of the extraction site. These differences are observed both between institutions and among surgeons within the same institution.

The objective of the present survey was to explore current surgical practices and preferences in performing RHC for colorectal cancer, with the aim of identifying opportunities for technical improvement and standardization.

## Materials and methods

### Type of study

The present study was a cross-sectional web-based survey composed of 31 questions on practices for performing RHC. The survey complied with the E-Surveys Checklist for Reporting Results of Internet [6, 7] and was published online using Google Form (Google, California, US). The survey was accessible from the 05.11.2023 to the 15.01.2024. The study did not require ethical clearance.

### Population

The survey was circulated on social media, such as Linkedin and Twitter, and was also made accessible by personal invitations through e-mails and messaging softwares. Target participants were general and/or colorectal surgeons practicing RHC.

### Variables of interest

The survey contained 31 questions, with both semi-quantitative and qualitative aspects. The survey investigated the demographics of participants (questions 1–7), their surgical experience in terms of RHC (questions 8–9), their preferred surgical approach for performing RHC (questions 10–13), the pre-operative preparation for patients undergoing RHC (14–16), the participants’ preferred technique for mobilizing the right colon (question 17), for performing lymphadenectomy (questions 18–21 and 25) and for vascular control (questions 22–23), whether participants performed complete mesocolic excision (CME)(question 24), the participants’ preferred technique for performing the ileo-colic anastomosis (questions 25–29), their preferred extraction site (question 30) and if they include patients in an enhanced recovery after surgery (ERAS) program or not (question 31).

## Results

### Participants

A total of 368 surgeons from 48 countries participated in the survey conducted between November 2023 and January 2024. Among them, 245 (66.6%) were general surgeons and 98 (26.6%) were specialized in colorectal surgery. Most respondents (304; 82.6%) were employed as consultant surgeons, while 64 (17.4%) were surgical residents/fellows. The median participant age was 38 years (range: 21–67), with 305 respondents (82.9%) identifying as male. Participants practiced in public academic hospitals (218; 59.2%), public non-academic hospitals (101; 27.4%), and private clinics (49; 13.3%). Regarding procedural experience, 167 participants (45.4%) had performed between 1 and 50 RHC, 75 (20.4%) had performed between 50 and 100, and 126 (34.2%) had performed over 100 RHC during their careers. In terms of annual volume, 147 participants (39.9%) performed between 1 and 10 RHC per year, 128 (34.8%) between 11 and 20, 58 (15.8%) between 20 and 30, and 35 (9.5%) more than 30 RHC per year.

### Perioperative care

Most respondents (348; 94.6%) routinely administered preoperative antibiotics. OA, either alone or in combination with intravenous antibiotics, were used by 89 participants (24.2%), whereas intravenous antibiotics were used by 259 participants (70.4%). Twenty participants (5.4%) reported not administering antibiotics prior to performing RHC. Mechanical bowel preparation (MBP) was routinely performed by 159 respondents (43.2%), with 39 (10.6%) combining it with OA. Enhanced recovery after surgery (ERAS) protocols were routinely employed by 249 out of 296 respondents (84.1%).

### Surgical approach

Laparoscopy was the preferred surgical approach for performing RHC among 254 participants (69.0%), followed by open surgery (82; 22.3%) and robotic surgery (32; 8.7%). A total of 173 respondents (47%) performed minimally invasive RHC in over 90% of their cases, while the remaining participants were almost evenly distributed across the other usage groups (< 25%, 25–50%, 50–75%, and 75–90%). Eighteen participants (4.9%) did not perform minimally invasive RHC. Among 349 respondents who reported performing laparoscopic RHC, multiport laparoscopy was preferred by 344 (98.6%) and single-port laparoscopy by 5 (1.4%).

Robotic RHC was performed by 148 participants (40.2%), with preferred platforms being the Da Vinci Xi (126; 85.1%), Da Vinci X (13; 8.8%), Da Vinci Si (3; 2.0%), CMR Versius (3; 2.0%), Medtronic HUGO (2; 1.4%), and Da Vinci SP (1; 0.7%).

### Colonic mobilization

Among the 350 participants performing minimally invasive RHC, the medial-to-lateral approach was most commonly used (288 participants; 82.3%), followed by the bottom-up/infra-mesenteric (45 participants; 12.9%) and the lateral-to-medial (17 participants; 4.9%) approaches.

### Lymphadenectomy

*Laparoscopic RHC*: Of 349 participants, 300 (86.0%) performed intra-corporeal lymphadenectomy at the ileo-colic pedicle, 38 (10.9%) performed it extra-corporeally in selected cases, and 11 (3.2%) in all cases. For middle colic vessels, intra-corporeal lymphadenectomy was used by 270 (77.4%), extra-corporeal in selected cases by 69 (19.8%), and always extra-corporeal by 10 (2.9%).

*Robotic RHC*: Of 151 participants, 140 (92.7%) performed intra-corporeal lymphadenectomy at the ileo-colic pedicle, 9 (6%) extra-corporeally in selected cases, and 2 (1.3%) in all cases. For middle colic vessels, intra-corporeal lymphadenectomy was performed by 147 (92.5%), and extra-corporeal in selected cases by 11 (7.5%).

### Lymph node labeling

For oncologic resections, lymph nodes can be labeled according to their anatomical location. Lymph node stations were routinely labeled by 43 participants (11.7%) and selectively labeled by 87 (23.6%). Most participants (236 participants; 64.7%) did not label lymph node stations when sending the operative specimen for anatomopathological examination.

### Complete mesocolic excision (CME)

CME was routinely performed by 152 participants (41.3%), selectively by 158 (42.9%), and not performed by 58 (15.8%).

### Vascular control

Among 350 participants performing minimally invasive RHC, arterial control was most commonly achieved using Hem-o-lok clips (252 participants; 72%), followed by metal clips (55 participants; 15.7%), energy sealing devices (25 participants; 7.1%), and vascular staplers (18 participants; 5.1%). For venous control, Hem-o-lok clips were also preferred (239 participants; 68.3%), followed by metal clips (54 participants; 15.4%), sealing devices (37 participants; 10.6%), and vascular staplers (20 participants; 5.7%).

### Anastomosis

*Fluorescence angiography*: Fluorescence angiography was routinely used by 147 participants (39.9%) to assess bowel perfusion, whereas 221 participants (60.1%) did not use it.

*Design*: The most common configuration for ileo-colic anastomosis was side-to-side isoperistaltic (246; 66.8%), followed by side-to-side antisperistaltic (77 participants; 21.0%), end-to-side (25 participants; 6.8%), and end-to-end (20 participants; 5.4%).

*Open RHC*: Among 368 participants, stapled anastomosis was more frequently performed (189 participants; 51.4%) than handsewn techniques (171 participants; 46.5%). When handsewn, the anastomosis was performed using running (138 participants; 80.7%) or interrupted sutures (33 participants; 19.3%). Single-layer sutures were preferred by 68 participants (39.8%), while 103 (60.2%) used a double-layer technique. When stapled, partial stapling with closure of the enterotomy using sutures was preferred (112 participants; 59.3%) over complete stapling (77 participants; 40.7%).

*Laparoscopic RHC*: Of 347 respondents performing laparoscopic RHC, 177 (51.0%) preferred extra-corporeal anastomosis (ECA), while 165 (47.6%) opted for intra-corporeal anastomosis. Among extra-corporeal handsewn procedures, running sutures were used by 67 (81.7%), and interrupted sutures by 15 (18.3%). Single-layer closure was used by 35 (42.7%) and double-layer by 47 (57.3%). Among stapled anastomoses, complete stapling was preferred by 59 (62.1%), and partial stapling by 36 (37.9%). In intra-corporeal handsewn cases, 16 (84.2%) used running sutures and 3 (15.8%) used interrupted. Single-layer was chosen by 11 (53.2%) and double-layer by 7 (36.8%). Among stapled intra-corporeal anastomoses (ICA), partial stapling was strongly preferred (135; 92.5%), with complete stapling reported by 11 (7.5%).

*Robotic RHC*: Of 146 respondents performing robotic RHC, 121 (82.9%) preferred intra-corporeal anastomosis, and 24 (16.4%) extra-corporeal. Among extra-corporeal handsewn anastomoses, running sutures were used by 8 (64.3%), interrupted sutures by 5 (35.7%), single-layer by 5 (35.7%), and double-layer by 8 (64.3%). Among stapled procedures, complete stapling was preferred by 7 (63.6%) and partial stapling by 4 (36.4%). For intra-corporeal handsewn anastomosis, 18 (81.8%) used running sutures, 4 (18.2%) used interrupted, 8 (36.4%) preferred single-layer, and 14 (63.6%) used double-layer closure. Among stapled intra-corporeal procedures, partial stapling was the preferred method (98 participants; 99.0%), while complete stapling was used by only one participant (1.0%).

### Extraction site

Preferred extraction sites RHC were the Pfannenstiel incision (171 participants; 46.5%), midline incision (148 participants; 40.2%), transverse incision (36; 12.8%), and natural orifice specimen extraction (NOSE) (reported by 2 participants, 0.5%). A Pfannenstiel incision was the preferred extraction site for 75% of surgeons when performing robotic RHC, and by 46% of surgeons when performing laparoscopic RHC.

## Discussion

Right hemicolectomy (RHC) is a frequently performed procedure, with reported anastomotic leak rates reaching up to 8.1% [8]. Despite this, there is still a significant variability in surgical practice, as individual surgeons tend to adopt their own techniques and preferences.

Although minimally invasive techniques have demonstrated the superiority over open approaches in terms of short-term outcomes [9], our survey reveals that 22.3% of respondents still perform RHC via open surgery as a preferred approach which is close to percentage found in some nationwide cohort studies [10]. This highlights a persistent gap between evidence-based recommendations and real-world practice.

The use of MBP and OA before RHC remains controversial. Some studies have failed to demonstrate a clear benefit of MBP in laparoscopic colectomy [11], while others, including the ORALEV trial, support the use of OA to improve surgical outcomes [6]. Furthermore, a recent analysis suggests that combining MBP and OA may reduce the incidence of surgical site infections (SSI) [12]. In our cohort, only 24.2% of surgeons reported using preoperative OA, and 43.2% reported using MBP, reflecting this ongoing uncertainty. And more concerning, a significant proportion of surgeons did not use any preoperative antibiotics at all.

Furthermore, the literature support the benefits of ICA versus ECA [13]. Indeed, some studies suggest that ICA leads to faster recovery and a reduced risk of paralytic ileus [14, 15], while others, such as the MIRCAST trial, report no significant difference in postoperative outcomes between ICA and ECA [16]. In our survey, surgeons performing robotic RHC overwhelmingly preferred stapled ICA, yet only 8.7% of respondents reported using a robotic approach, despite the perceived advantages of this technique for performing fully minimally invasive RHC [17]. It is obvious that robotic platform favor ICA but remains still a minority, for example in Denmark between 2015 and 2018, only 9.5% of RHC were done using robotic platform [10].

In our survey, the Pfannenstiel incision was the most frequently used extraction site (46.5%), which shows understanding that midline extraction should be avoided to diminish incisional hernias [18, 19]. The literature also shows evidence that suggests ICA may be associated with improved outcomes, including reduced extraction site complications [20, 21]. One might assume that such variability arises from differing levels of surgical training. However, when conducting a comparable analysis restricted to board-certified colorectal surgeons, these discrepancies persist. Specifically, 81.6% report using laparoscopy, with the medial-to-lateral dissection remaining the most commonly adopted approach. Regarding anastomosis, it is preferentially performed extracorporeally (55.1%) and using mechanical stapling (74.5%). Finally, within this subgroup, the midline incision is the most frequently selected access route, employed in 46.9% of cases. The only notable distinction observed among board-certified colorectal surgeons is the marked reduction in the use of the open approach for right hemicolectomy. Although robotic systems provide superior dexterity and visualization that can facilitate a fully minimally invasive approach, their widespread adoption is still limited by restricted access, high costs, and the absence of standardized training programs [22, 23]. From a practical standpoint, further clarification of these aspects along with the establishment of evidence-based technical guidelines and structured training pathway appears essential to ensure the safe and consistent implementation of minimally invasive and robotic RHC worldwide. Future collaborative efforts and prospective studies should aim to address these uncertainties to promote technical standardization and optimize patient outcomes.

Despite recent progress in surgical innovation, several uncertainties persist regarding the optimal conduct and implementation of minimally invasive right hemicolectomy. Significant variability remains in key technical aspects, including the extent of lymphadenectomy (D2 vs. D3), the systematic use of complete mesocolic excision (CME), and the preferred type and configuration of ileocolic anastomosis [24].

A limitation of this survey lies in its design. For instance, data collection was based on self-reported practices, which may introduce bias. Additionally, the absence of outcome correlation prevents establishing causality between reported techniques and postoperative complications. Well-designed randomized controlled trials are needed to rigorously compare surgical approaches, particularly regarding the clinical impact of ICA versus ECA, robotic versus laparoscopic RHC, and the role of bowel preparation strategies.

## Conclusion

This international survey highlights substantial variability in the surgical approach to RHC, particularly regarding lymphadenectomy, anastomotic techniques, and extraction site preferences. While laparoscopy is widely adopted, a fully minimally invasive approach—characterized by intracorporeal anastomosis and optimized extraction sites—remains underutilized. Robotic platforms appear to facilitate such approaches but are not yet widespread. The heterogeneity observed underscores the need for standardization based on high-quality evidence. Future prospective studies should aim to clarify the clinical impact of key technical choices, including the type of anastomosis, the use of robotic assistance, and perioperative preparation strategies, to promote best practices and improve patient outcomes.

## Supplementary Information

Below is the link to the electronic supplementary material.

Supplementary file1 (XLSX 82 kb)

## Abbreviations

RHC: Right hemicolectomy
ICA: Intracorporeal anastomosis
ECA: Extracorporeal anastomosis
OA: Oral antibiotics
MBP: Mechanical bowel preparation
